# Exact Probability Distribution for the ROC Area under Curve

**DOI:** 10.3390/cancers15061788

**Published:** 2023-03-15

**Authors:** Joakim Ekström, Jim Åkerrén Ögren, Tobias Sjöblom

**Affiliations:** Department of Immunology, Genetics, Pathology, Uppsala University, Rudbecklaboratoriet, 752 57 Uppsala, Sweden

**Keywords:** receiver operating characteristic, AUC-value distribution function, AUC *p*-value, exact test

## Abstract

**Simple Summary:**

This contribution allows for the computation of exact *p*-values and for conducting accurate statistical hypothesis tests of ROC AUC-values. As a result, the development of diagnostic tests is facilitated. This work is illustrated via simulated data and through the development of proteomic blood biomarkers for the early detection of cancer.

**Abstract:**

The Receiver Operating Characteristic (ROC) is a de facto standard for determining the accuracy of in vitro diagnostic (IVD) medical devices, and thus the exactness in its probability distribution is crucial toward accurate statistical inference. We show the exact probability distribution of the ROC AUC-value, hence exact critical values and *p*-values are readily obtained. Because the exact calculations are computationally intense, we demonstrate a method of geometric interpolation, which is exact in a special case but generally an approximation, vastly increasing computational speeds. The method is illustrated through open access data, demonstrating superiority of 26 composite biomarkers relative to a predicate device. Especially under correction for testing of multiple hypotheses, traditional asymptotic approximations are encumbered by considerable imprecision, adversely affecting IVD device development. The ability to obtain exact *p*-values will allow more efficient IVD device development.

## 1. Background

The ROC concept [1] has become a de facto standard for determining the accuracy of binary predictors within many areas, such as IVD medical devices [2,3,4]. In order to provide a regulator with valid scientific evidence supporting the conclusion that there is reasonable assurance that an IVD device is safe and effective [5], it is highly valuable to demonstrate that the ROC Area Under Curve (AUC) value of the novel IVD device is non-inferior relative to a predicate device. Because the ROC-curve and the AUC-value is subject to randomness due to sources of error including sampling error and measurement imprecision, statistical hypothesis tests are necessary.

The motivation for this work, specifically, is our effort to develop biomarkers for the early detection of cancer. Because of combinatory proliferation when composite biomarkers are generated, the correction for testing multiple hypotheses is often severe, yielding critical values that are situated in the far tails of the AUC-value probability distribution. It is a well-known phenomenon that the tails of probability distributions are particularly sensitive to inaccuracies; hence, exactness in the AUC-value probability distribution is crucial toward accurate statistical inference.

The term ROC was introduced by Ref. [6] and the phrase AUC was used by Ref. [7]. While a complete literature review is beyond the scope of this article, a summary of approaches used to approximate the AUC-value probability distribution is warranted and follows in this paragraph and the next. It was shown [8] that when computed by the trapezoidal rule, the trapezoidal AUC-value equals a constant times the Mann–Whitney U statistic, which is asymptotically normal [9]. Refs. [10,11,12,13,14] use this rationale toward a normal distribution approximation, with variance estimates under a range of assumptions. Under a normal distribution assumption, a confidence interval for the AUC-value can extend above 1, and therefore transformations have been proposed [14,15,16] to ensure that the confidence interval is contained in the interval [0, 1].

The method of empirical likelihood was proposed by Ref. [17], yielding an empirical log-likelihood ratio that is asymptotically $\chi^{2}$-distributed. A distinct approach is to estimate the probability distributions of the cases and controls by kernel smoothing methods  [15,18], where the obtained AUC-value is either approximately normally distributed per the aforementioned rationale, or can be estimated by bootstrap, jackknife, or similar computer intensive methods as proposed by Ref. [19]. Outright Monte Carlo-simulation of AUC-values has also been proposed [20]. Parametric estimation of the probability distributions of the cases and controls has been discussed [21,22], yielding Wald and likelihood ratio statistics, which are both asymptotically $\chi^{2}$-distributed. Reviews of note are Chapter 1 of Ref. [23], which discuss the development of the ROC concept, and Refs. [24,25], which discuss ROC statistics and their distributions in detail. A large number of approximate methods is compared by Ref. [19] under a range of conditions, showing through simulation, e.g., that asymptotic approximations are particularly challenging when the underlying AUC-value is above 0.85 due to a slow rate of convergence.

This article is structured as per the following. In three subsections, Section 2 discusses obtaining the AUC-value probabilities through exact computation, Monte Carlo-simulation, and through geometric interpolation between the aforementioned two. Section 3 discusses employing the AUC-value probability distribution for statistical hypothesis testing purposes. Section 4 illustrates the methods through simulated and real data, and Section 5 and Section 6 contain the discussion and conclusions.

## 2. Probability Distribution of the AUC-Value

### 2.1. Exact Computation via Order Statistics

This subsection derives the exact AUC-value probability distribution under the assumption of observed values of the True Positives (TPs) and True Negatives (TNs) that each are independent and identically distributed, *iid* with probability distribution functions denoted as *F* and *G*, respectively.

Because the ROC-curve is determined by the ranks of the observed values of the TPs and TNs, the probability of an AUC-value can be computed through order statistics. In general, suppose there are *n* observed values of the TPs, $x_{1},\cdots ,x_{n}$, and *m* observed values of the TNs, $y_{1},\cdots ,y_{m}$, and the two have probability density functions *f* and *g*, respectively, then under the *iid* assumption the order statistic joint probability density function, η, presuming they are indexed such that $y_{1}\leq \cdots \leq y_{m}$ and $x_{1}\leq \cdots \leq x_{n}$, equals

$$\left\{\begin{array}{cc} m!n!g\left(y_{1}\right)\cdots g\left(y_{m}\right)f\left(x_{1}\right)\cdots f\left(x_{n}\right) & \mathrm{if}\;y_{1}\leq \cdots \leq y_{m}\;\mathrm{and}\;x_{1}\leq \cdots \leq x_{n},\\ 0 & \mathrm{otherwise}. \end{array}\right.$$

Because each ROC-curve is determined completely by the ranks of the observed values of the TPs and TNs, the probability that a given ROC-curve will manifest itself can thus be obtained through integration of the joint probability density function.

For example, the ROC-curve that has an AUC-value equal to unity corresponds to observing values of the TPs and TNs such that $y_{m}\leq x_{1}$, and under the *iid* assumption and existence of probability density functions, the probability of the ROC-curve equals

$$\int_{-\infty }^{\infty }\cdots \int_{-\infty }^{x_{2}}\int_{-\infty }^{x_{1}}\cdots \int_{-\infty }^{y_{2}}\eta \;dy_{1}\cdots dy_{m}dx_{1}\cdots dx_{n},$$

which after application of the chain rule can be shortened to

$$n\int_{-\infty }^{\infty }{\left(1-F\left(x_{1}\right)\right)}^{n-1}f\left(x_{1}\right)G{\left(x_{1}\right)}^{m}\;dx_{1}.$$

For another example, the ROC-curve that has an AUC-value equal to 1−1/mn, i.e., the second highest AUC-value possible, corresponds to observing values of the TPs and TNs such that $y_{m-1}\leq x_{1}\leq y_{m}\leq x_{2}$, and under the same assumptions the probability of that ROC-curve equals

$$mn\left(n-1\right)\int_{-\infty }^{\infty }\int_{-\infty }^{x_{2}}\int_{-\infty }^{y_{m}}{\left(1-F\left(x_{2}\right)\right)}^{n-2}f\left(x_{2}\right)f\left(x_{1}\right)g\left(y_{m}\right)G{\left(x_{1}\right)}^{m-1}\;dx_{1}dy_{m}dx_{2}.$$

Continuing to the example of the ROC-curve with AUC-value 1−2/mn, the reader will note that there are two distinct ROC-curves that produce the AUC-value, one that corresponds to observing values of TPs and TNs such that $y_{m-1}\leq x_{1}\leq x_{2}\leq y_{m}\leq x_{3}$ and the other such that $y_{m-2}\leq x_{1}\leq y_{m-1}\leq y_{m}\leq x_{2}$. Because the two ROC-curves are mutually exclusive, the probability of observing TPs and TNs such that the AUC-value equals 1−2/mn is equal to the sum of the probabilities of those two ROC-curves. Generally, distinct ROC-curves are mutually exclusive and consequently the probability of a given AUC-value equals the sum of the probabilities of all ROC-curves that produce the given AUC-value.

In practice, numerical computation requires explicit probability distribution functions *F* and *G*. A common choice of probability distribution functions is two normal distributions with equal variance and some difference in their means [25], sometimes referred to as binormal. Since many common parametric choices of probability distributions are quite smooth, numerical integration using the trapezoid method is typically accurate and also fast in its algorithmic implementation [26].

**Figure 1:**
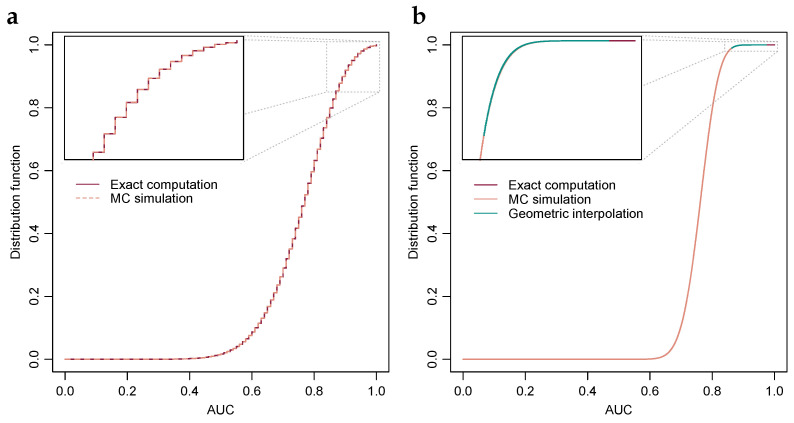

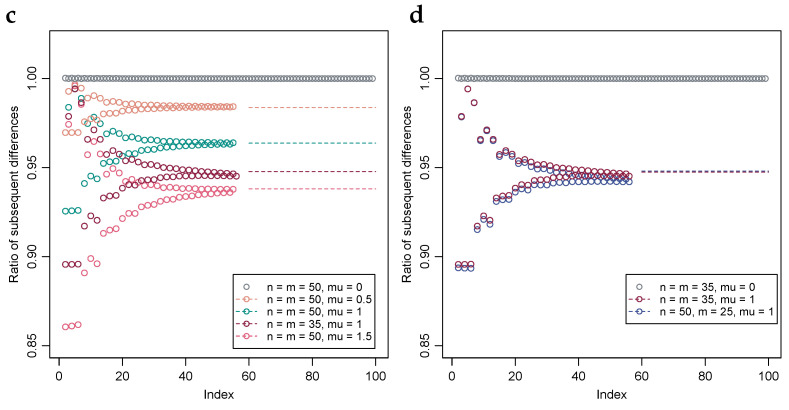
Exact probability distributions of the AUC-value under a range of parameters. (**a**) Exact computation and Monte Carlo estimation of the AUC-value distribution when TPs and TNs are 10 each and *iid* with normal distributions of equal variance and unit difference in mean. Inset, top 10 percentiles. (**b**) Exact computation and MC estimation of the AUC-value distribution when TPs and TNs are 50 each and *iid* normal with equal variance and unit difference in means. Geometric interpolation between exact computation and the MC estimated 99th percentile is included. Inset, top percentile. (**c**) Stability of the ratio of subsequent differences under a range of parameters, i.e., $\left(\Delta x_{i}/\Delta x_{i-1}\right)/\left(\Delta k_{i}/\Delta k_{i-1}\right)$, with solution through the bisection method included in dashed lines. (**d**) Plot similar to Subfigure (**c**), albeit with numbers of TPs and TNs selected such that their products are approximately equal, with two selections having equal numbers of TPs and TNs and the third twice the TPs relative to TNs.

The probability distribution function of the AUC-value can be determined by computing the probability for each of the AUC-values 0,1/mn,2/mn,⋯,1; for an illustration, see Figure 1a. However, the number of ROC-curves that produce a given AUC-value tends to be large in many situations, particularly when the AUC-value is around the centre of the unit interval, and consequently determining the whole AUC-value probability distribution function through this method is in many instances computationally impractical.

### 2.2. Monte Carlo-Simulation of the AUC-Value Probabilities

When estimated through *iid* sampling, by the strong law of large numbers the empirical distribution function converges point-wise, with probability one, to the distribution function from which the observations were drawn. The result can be strengthened further; through the Glivenko–Cantelli lemma the convergence is uniform and through Donsker’s theorem the normalized difference converges in distribution to a Gaussian process [27]. Because of its desirable asymptotic properties, and properties such as simplicity of computation, the empirical distribution function is commonly utilized for estimation of the distribution functions through Monte Carlo-simulation.

Percentiles can be estimated through the empirical distribution function by taking the infimum of the superlevel set, i.e., if α is a number in the unit interval then the 100α-percentile is estimated through $\inf\{x:{\hat{F}}_{k}\left(x\right)\geq \alpha \}$, where ${\hat{F}}_{k}$ denotes the empirical distribution function at sample size *k*. In a common algorithmic implementation, determining the empirical distribution function and the infimum of the superlevel set amounts to sorting the observed values and selecting the value that is the 100α percent largest. Through the aforementioned beneficial asymptotic properties of the empirical distribution function, the percentile estimate obtains many desirable properties, however if the distribution function is constant in an interval then the percentile estimate obtains a discontinuity when viewed as a function of α. Further, even if the distribution function is strictly increasing, a relatively shallow slope of the distribution function yields the practical problem of a slow rate of convergence.

When correction for testing of multiple hypotheses is applied, which is common in for instance biomarker discovery, the critical values to be estimated are typically situated in the far tail of the probability distribution, where the distribution function often has a shallow slope. As an illustration, if the type-one error probability subsequent to correction for testing of multiple hypotheses equals $10^{-10}$, then the Monte Carlo-simulation estimate of the corresponding critical value is equal to the maximum AUC-value simulated for all simulations that are constituted of $10^{10}$ or fewer simulated AUC-values. However, in contrast, as discussed in Section 2.1, computing the exact probability of AUC-values is relatively quick when the AUC-values are close to the extremities of the unit interval. A pragmatic compromise is to employ exact computation in the far tails and use Monte Carlo-simulation in the body of the AUC-value distribution.

### 2.3. Geometric Interpolation

As discussed, the number of AUC-value probabilities that can be computed exactly is in practice limited by the availability of computational resources. At the time of writing, we use a computer with an Intel (Santa Clara, CA, USA) Xeon W-2123 CPU, having 8 cores running at 3.6 GHz, and 48 GB RAM, and computing 50 consecutive AUC-value probabilities requires less than 2 h while computing 60 AUC-value probabilities requires about 13 h. We have observed that each subsequent AUC-value probability requires 22% additional time relative to the preceding AUC-value probability, thus yielding an exponential growth in the amount of time required as the number of consecutive AUC-value probabilities computed increases.

Ideally, the sum of the AUC-value probabilities, computed consecutively from zero or one, is such that the corresponding percentile is suitable for estimation through Monte Carlo-simulation. However, in many instances the sum of the computed AUC-value probabilities is materially smaller than would be desired vis-à-vis percentile estimation through Monte Carlo-simulation. Consequently, the limitation of computational resources effectively yields a gap between the sum of the computed AUC-value probabilities and the largest value deemed suitable for percentile estimation through Monte Carlo-simulation. In this instance, we have observed a perceived stability of ratios of subsequent differences of AUC-value probabilities, arising from a special case, and exploited it toward bridging the aforementioned gap through geometric interpolation.

Denote by i=0,1,2,3,⋯ the sequence of AUC-values 1,1−1/nm,1−2/nm,1−3/nm,⋯, where *n* and *m* are, as in Section 2.1, the number of observed values of TPs and TNs, respectively. Suppose the observed values of TPs are *iid* with probability distribution function *F* and the observed values of TNs are *iid* with probability distribution function *G*, and denote by $x_{0},x_{1},x_{2},x_{3},\cdots$ the probabilities that the AUC-value attains 1,1−1/nm,1−2/nm,1−3/nm,⋯.

**Figure 2:**
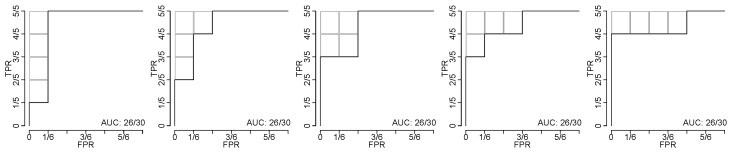
Five ROC-curves yielding the same AUC-value. Illustration of the five distinct ROC-curves that yield the AUC-value 26/30 when the number of TPs and TNs are n=5 and m=6, respectively. The gray boxes illustrate the subtracted four rectangles each with area 1/nm; hence 1−4/nm=26/30.

Consider firstly the special case when F=G, i.e., the observed values of the TPs and TNs follow the same probability distribution. Then the expressions of Section 2.1 simplify so that the probability of each ROC-curve equals n!m!/(n+m)!, and therefore the probability of an AUC-value is determined by the number of distinct ROC-curves that yield the AUC-value. Consequently, in this special case it holds that $x_{i}=k_{i}n!m!/\left(n+m\right)!$, where $k_{0},k_{1},k_{2},k_{3},\cdots$ denotes the number of distinct ROC-curves that yield the AUC-values 1,1−1/nm,1−2/nm,1−3/nm,⋯. Figure 2 illustrates the number of ROC-curves that yield the AUC-value 26/30 when n=5 and m=6; in the example $k_{4}=5$.

Let Δ denote the difference operator, $\Delta x_{i}=x_{i}-x_{i-1}$. The present authors have observed that, in the top percentile of the probability distribution, the ratio of subsequent differences $\Delta x_{i}/\Delta x_{i-1}$ is well approximated by $C\Delta k_{i}/\Delta k_{i-1}$ where *C* is a constant. As discussed, this is in the special case when F=G equality holds with C=1. See Figure 1c,d for a numerical example with a selection of values of *n* and *m*, and where *F* and *G* are each normal distributions with equal variance and a selection of differences in their means. Rearranging terms yields the approximation

$$x_{i}\approx x_{i-1}+C\;\frac{\Delta k_{i}\Delta x_{i-1}}{\Delta k_{i-1}},$$

which can be employed to bridge the gap between the exact computations discussed in Section 2.1 and the Monte Carlo-simulations discussed in Section 2.2.

An estimate of *C* in the above approximation can be obtained by solving for equality between the Monte Carlo estimate of the, say, 99.9th percentile and the same percentile when estimated through exact computation and geometric interpolation using the above approximation. For example, the bisection method is a simple yet effective method to solve for *C*, thus obtaining an estimate using the aforementioned equality [26]. The reader will note that there will be division by zero in the above approximation when $\Delta k_{i-1}=0$, i.e., $k_{i-1}=k_{i-2}$, however the authors have only seen this occur when i=2 or in some instances when i≈nm/2 and those instances do not constitute the typical intervals of application of the approximation, cf. Figure 1b.

**Figure 3:**
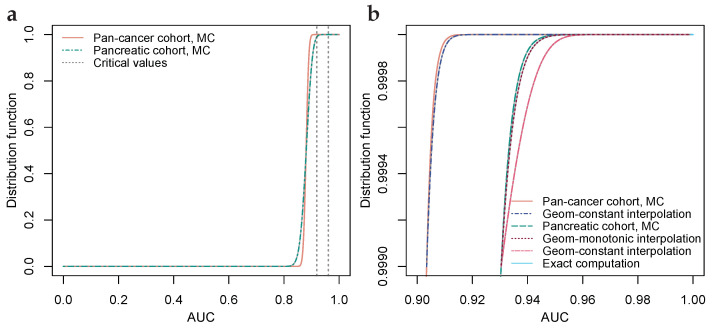

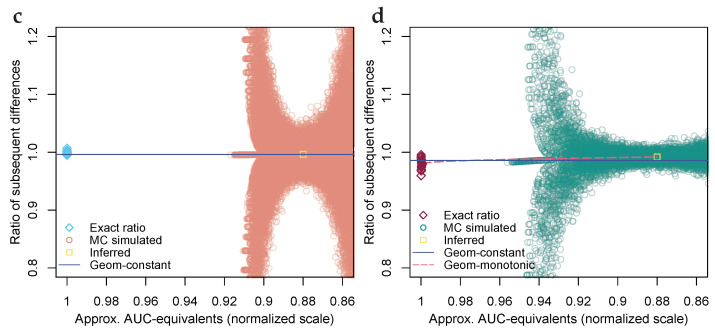
Geometric interpolation for cohorts with balanced and highly unbalanced numbers of TPs and TNs. (**a**) Monte Carlo-simulated AUC-value distribution functions for biomarkers on par with benchmark predicate FIT, with numbers of TPs and TNs 1004 and 800 (pan-cancer) and 93 and 800 (pancreatic). Critical values at 99% significance level, Bonferroni corrected for simultaneous testing of 53,352 hypotheses. (**b**) Upper 99.9th percentile of (**a**), with MC-simulation and exact computation bridged by geometric interpolations. For a pancreatic cohort, an alternative interpolation with a monotonically increasing geometric coefficient is included. (**c**) Ratio of subsequent differences for pan-cancer cohort, with a ratio inferred from the median of the S-shaped distribution function at AUC 0.88. (**d**) Ratio of subsequent differences for the pancreatic cohort, with a non-constant geometric coefficient, connecting the exactly computed ratios with the inferred ratio through a monotonically increasing positive exponent function.

While analyzing the dataset of Ref. [28], see Figure 3, we noted that the aforementioned ratio is less stable when the numbers of TPs and TNs are highly unbalanced. In Figure 3d, where the balance of TPs to TNs for the pancreatic cohort is approximately 1:9, a slightly positive trend can be visually perceived, and when a constant is assumed, the distribution function will, seen from the right, firstly drop too quickly and then too slowly, see Figure 3b. When an approximation is unsatisfactory, common approaches are to either use a more sophisticated approximation or to lessen the use of the approximation. Figure 3d shows an approximation using a monotonically increasing function, an exponent function, that connects the AUC-value probabilities with the AUC-value probability at the median, which is inferred from the curvature of the S-shape yielding $\Delta x_{i}/\Delta x_{i-1}=1$. Lessening the use of the approximation is achieved by shortening the gap to be bridged; computing more exact AUC-value probabilities and estimating a higher percentile through Monte Carlo-simulation.

Determining the numbers $k_{0},k_{1},k_{2},\cdots$, i.e., the numbers of distinct ROC-curves that yield an AUC-value, see Figure 2 for an illustrative example, can be achieved through the following recursion, which is stylized in pseudo-code in Algorithm 1.
**Algorithm 1:** Recursive algorithm returning the number of ROC curves that yield an input AUC-value1:**procedure** RecursFcn (*x*, max_dim1, max_dim2)2:    **if** x<0 **or** max_dim2<03:        **return** 04:    5:    **if** max_dim1==16:        **if** x<=max_dim27:            **return** 18:        **else**9:            **return** 010:    11:    **if** x==012:       **return** 113:    14:    **return** RecursFcn(*x*, max_dim1−1, max_dim2)15:               +RecursFcn(x−max_dim1, max_dim1, max_dim2−1)16:**end procedure**

The recursive algorithm employs the recursive equality that is described in the following. Denote by (u·v) the number of permutations in which *u* rectangles, each with area 1/nm cf. Figure 2, can be subtracted under a maximum of *v* rectangle side lengths. For instance, the side length may be taken along the horizontal axis, cf. Figure 2, where the maximum number of horizontal side lengths is 6. The number of permutations (u·v) can be decomposed into two parts as per the equality    

(u·v)=(u·v−1)+(u−v·v).

The first part of the decomposition are the permutations that use only v−1 side lengths, and the second part are the permutations that use all *v* side lengths, which therefore equals the number of permutations (u−v·v).

The recursion terminates under the conditions (u·v)=0 for all u<0, i.e., there are nil permutations in which a negative number of rectangles can be subtracted, (u·1)=1, i.e., there is one permutation in which any number of rectangles can be subtracted under a maximum of only one side length, and (0·v)=1, i.e., there is one permutation in which nil rectangles can be subtracted under any maximum of rectangle side lengths. Further, a condition on the maximum of the number of side lengths along the axis perpendicular relative to the axis thus discussed is implemented, denoted by *max_dim2* in the pseudo-code. While the authors view it as possible that a closed form expression for the numbers $k_{i}$, i=0,1,⋯,nm, exists, we have at the time of writing not been able to derive such; hence the recursive algorithm.

## 3. Statistical Hypothesis Testing of AUC-Values

Because the AUC-value is a number in the unit interval, a one-sided acceptance region with type-one error probability α is constructed through the interval [0,c] where *c* is the critical value satisfying P(X>c)=α and *X* is the random test variable under the null-hypothesis [29]. In applications such as biomarker discovery, it is common to simultaneously consider numerous binary predictors. In particular, systematically forming composite biomarkers from constituent measurands often causes a combinatory proliferation, and thus AUC-values for millions of composite biomarkers may be simultaneously considered. As a result of correction for testing of multiple hypotheses through Bonferroni-correction or other methods, the type-one error probability, α, is commonly small. For a numerical example, a statistical significance level of 99% and $10^{8}$ composite biomarkers yields a Bonferroni-corrected α-value of $10^{-10}$.

With AUC-values, estimation of the critical value through outright Monte Carlo-simulation will be especially taxing because each AUC-value requires simulation of the observed values of TPs and TNs. Within the setting of a clinical trial, the numbers of TPs and TNs could be between 100 and 1000 each, and to obtain an estimate of a critical value at α equalling $10^{-10}$, at least $10^{10}+1$ AUC-value simulations are needed. Hence, to obtain the most coarse estimate of the critical value through Monte Carlo-simulation, several trillion random numbers are often needed, and to obtain a more precise estimate perhaps a quadrillion random numbers would be desired in the presently discussed numerical example.

Furthermore, when the purpose of the Monte Carlo-simulation is estimation of statistical power or experimental design optimization, then simulations will typically need to be performed under a range of design parameter values, which will greatly compound the difficulties. For a numerical indication, at the time of writing the authors are able to simulate $10^{10}$ AUC-values in $2.63\times 10^{6}$ s, or 30.4 days, at 400 cases and 400 controls using computationally optimized algorithms in R (version 3.3.2)  [30] and computer equipment as detailed in Section 2.3. Simulation across a range of sample sizes and possibly other design parameters would multiply the required time by several folds. Hence, statistical hypothesis testing of AUC-values is simple in principle, but the determination of the probability distribution tails is computationally challenging.

## 4. Examples

### 4.1. Illustration through Simulated Data

For illustrative purposes, a few numerical examples are provided. In these examples the observed values of TPs and TNs are each normally distributed with unit variance and some difference in their mean values. When the difference in means is taken to be equal to one, then the resulting AUC-value is on average about 0.76 with a standard deviation that depends largely on the number of TPs and TNs.

Figure 1a,b show probability distribution functions of the AUC-value when the differences in the means of the observed values of TPs and TNs are equal to one, and the number of TPs and TNs are n=m=10 and n=m=50, respectively. When the numbers of TNs and TPs are 10, then the AUC-value can attain 101 distinct values and consequently it is feasible to compute the probability of each AUC-value exactly, as per Section 2.1. In addition, estimates of the probabilities obtained through Monte Carlo-simulation, as per Section 2.2, are shown. Alignment between the two is visually evident.

In Figure 1b, the numbers of TNs and TPs are 50, and consequently the AUC-value can attain 2501 distinct values, and with the computers used at the time of writing computation of exact probabilities of all AUC-values, as per Section 2.1, it was deemed infeasible as it would require several years of computing time as per the observed exponential growth detailed in Section 2.3. The 57 AUC-value probabilities shown in Figure 1b have a sum of about $5.05\times 10^{-10}$ and the turquoise line shows geometric interpolation, as per Section 2.3, between the left-most AUC-value probability and the 99th percentile estimated through Monte Carlo-simulation. As examples, AUC-values 0.90, 0.95 and 0.99 have *p*-values $4.4\times 10^{-4}$, $4.3\times 10^{-7}$ and $8.5\times 10^{-16}$ respectively. Similarly, under Bonferroni-correction for $10^{8}$ hypotheses, the critical value for a one-sided test at the 99% significance level is 0.9816, i.e., if any of the observed AUC-values are greater than 0.9816 then the null-hypothesis is rejected; it seems improbable that the observed value is an observation from the hypothesized probability distribution.

Figure 1c,d illustrates the degree of stability of the ratios of subsequent probability differences normalized by the corresponding ratios of the differences of numbers of ROC-curves per AUC-values, i.e., the normalized ratios $\left(\Delta x_{i}/\Delta x_{i-1}\right)/\left(\Delta k_{i}/\Delta k_{i-1}\right)$, for the first 56 differences, which were the greatest number deemed feasible to compute when preparing the present example. As discussed in Section 2.3, when the TP and TN-distribution difference in means is zero, the ratio is identically equal to unity. The constant *C*, discussed in Section 2.3, is estimated using the bisection method, and is plotted in the figures in dashed lines. Alignments between the stabilizing ratios and the constants *C* estimated using the bisection method are visually evident.

Because the distribution function curve, cf. Figure 1b, possesses an S-shape, i.e., it first accelerates then decelerates, the ratio of subsequent probability differences is likely approximately constant only within an interval of limited length. However, as discussed in Section 2.3, the need to bridge exact computations and Monte Carlo-estimates is typically confined to the top or bottom percentile of the probability distribution, and within that finite region the approximation tends to be quite valid as illustrated in Figure 1c,d.

### 4.2. Illustration through Biomarker Data

Statistical hypothesis testing and determination of *p*-values of AUC-values under correction for multiple hypothesis testing is illustrated using the proteomic dataset of Ref. [28]. The data encompass 39 proteins measured across 1004 individuals newly diagnosed with cancer and 812 healthy controls. The cancer diagnoses are breast, colorectal, esophagus, liver, lung, ovary, pancreas, and stomach cancer. In addition, a ninth pan-cancer diagnosis is formed by merging the eight cancer diagnoses. It may be noted that the aforementioned data source did not include statistical hypothesis tests or *p*-values. For ovarian cancer, we chose to only include female healthy controls.

Composite biomarkers are formed as per Ref. [31]. From the 39 proteins, 741 composite biomarkers are formed by combining the proteins into pairs. The 741 are further multiplied by 8 as a result of allowing positive classification when one or both of the proteins are down-regulated and when either or both of the proteins are up or down-regulated. By testing for the 9 diagnoses, a total of (39choose2)×8×9= 53,352 composite biomarkers are simultaneously statistically hypothesis tested. *p*-values are adjusted as per the Bonferroni method. The null-hypothesis is that the biomarkers are on par with the colorectal cancer test FIT, which has an AUC-value of 0.88 ([32], p. 31). As a commonly used screening test, the stool-based test FIT is a direct predicate IVD device for the colorectal cancer cohort, and also a relevant benchmark for the other cohorts that lack regulatory approved screening predicate IVD devices [33]. Rejection of the null-hypothesis is interpreted as evidence that, sources of error notwithstanding, the biomarker has an AUC-value that is superior vis-à-vis the AUC-value of FIT.

For each of the 9 diagnoses, 58 AUC-value probabilities were computed exactly, i.e., 1,1−1/nm,1−2/nm,⋯,1−57/nm where *n* and *m* are the numbers of TPs and TNs, respectively. The nine probability distributions were then interpolated geometrically, as per Section 2.3, to the 99.99th percentiles, which were determined by Monte Carlo-simulation of $10^{8}$ AUC-values per diagnosis. The distinctly large Monte Carlo-simulation was used because some of the cohorts have highly unbalanced numbers of TPs to TNs, which affects the geometric interpolation as discussed in Section 2.3 and illustrated in Figure 3. Figure 3a shows distribution functions for the pan-cancer and pancreatic cohorts, where the difference in the number of TPs, 1004 versus 93, yields a lower critical value for the pan-cancer cohort relative to the pancreatic cohort. Figure 3c,d show geometric interpolation for the pan-cancer cohort and the pancreatic cohort, where the pan-cancer cohort possesses a balanced ratio of TPs to TNs (1004:812) and the pancreatic cohort possesses a highly unbalanced ratio of TPs to TNs (93:812) yielding a relatively better fit of the geometric constant interpolation for the pan-cancer cohort than for the pancreatic cohort (cf. Section 2.3). Using computer equipment as detailed in Section 2.3, computation of the 58 AUC-value probabilities required about 7.2 h per diagnosis, and the Monte Carlo-simulation required about 1.3 h per diagnosis. The probability distributions are available on GitHub as per the data availability statement.

In total, 26 cancer biomarkers were statistically significant at the 99%-level, meaning that they have an AUC-value that is higher than the highest AUC-value out of 53,352 biomarkers, which is on par with what FIT would reasonably produce. The interpretation is that those biomarkers are, sources of error notwithstanding, superior relative to FIT. In this instance, separation of training and validation data does not constitute an issue because every biomarker is tested statistically; i.e., no training is conducted.

**Figure 4:**
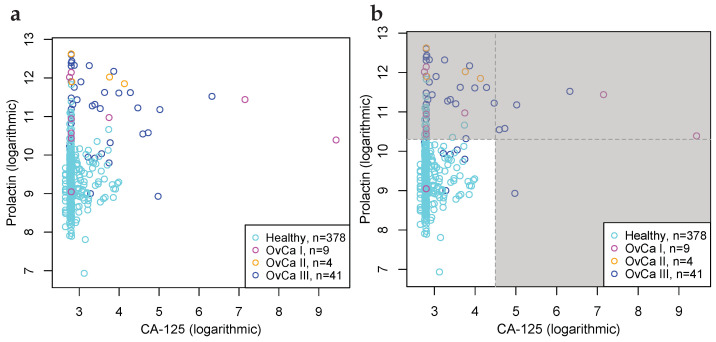

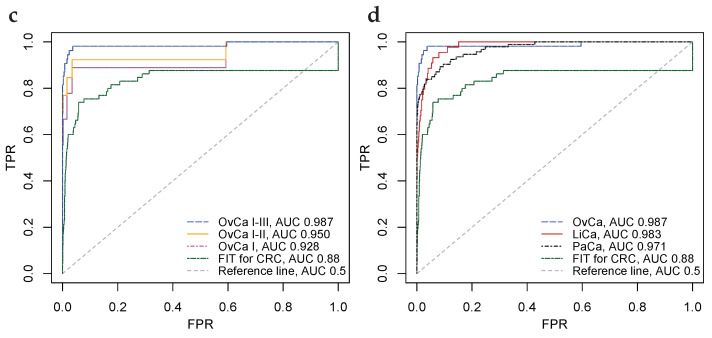
Biomarkers for the detection of ovarian, liver, and pancreatic cancers, that are superior vis-à-vis benchmark predicate device FIT at the 99% significance level. (**a**) Scatter plot of CancerSeek data, CA-125 and Prolactin, per OvCa stage (I, II and III) and female healthy controls. (**b**) Scatter plot similar to Subfigure (**a**), with cut-offs at 4.5 and 10.3; illustrating positive and negative classification regions. (**c**) ROC-curves for CA-125 and Prolactin as a biomarker for the detection of OvCa, per stages. Stronger biomarker signals for more advanced cancers are evident. For reference, FIT for CRC and the diagonal line is included. (**d**) ROC-curves for biomarkers for detection of OvCa (CA-125, Prolactin), LiCa (HGF, Osteopontin) and PaCa (CA 19-9, sErbB2). FIT and the diagonal line included for reference.

Table 1 shows Bonferroni corrected critical values relative to the null-hypothesis that the 53,352 biomarkers have an AUC-value on par with FIT. It is evident that tumour type cohorts that have larger sample sizes exhibit critical values that are lower than for cohorts that have relatively smaller sample sizes. Of the biomarkers tested, 26 are significantly better at the 99% level, than FIT, including 4 liver, 14 ovarian, and 8 pancreatic cancer biomarkers. Table 2 details those 26 biomarkers; which proteins they use, whether the proteins are up or down regulated, and whether up or down regulation of either protein or both is necessary for positive classification. A selection of significant biomarkers is graphically shown in Figure 4.

## 5. Discussion

Historically, AUC-value probability distributions have commonly been approximated via asymptotic properties or bootstrap-percentiles [19,24,25]; however the approach provides inadequate accuracy in the tails of the probability distribution where critical values under correction for multiple hypotheses are situated. With exact computation of AUC-value probabilities, impeccable precision is obtainable; permitting accurate statistical inference. In biomarker development, if critical values are too low, the type-one error probability will be too high; effectively yielding false expectations. If critical values are too high, then the type-two error probability will be too high; effectively barring identification of potentially valid biomarkers. Using the exact AUC-value probability distribution, the aforementioned risks are avoided, all while allowing for computation of exact *p*-values.

In order to provide reasonable assurance that a study has sufficiently large sample sizes to demonstrate a putative effect, experimental design optimization is warranted. The computational challenges inherent to Monte Carlo-simulation are greatly compounded when critical values under a multidimensional set of parameter values need to be estimated. With the proposed method of exact computation of AUC-value probabilities paired with Monte Carlo-simulations and geometric interpolation, a method is obtained that is both computationally feasible and relatively precise.

A disadvantage of the presently proposed approach using exact computation of AUC-value probabilities is that the method is relatively computationally intense. Historically, computers have become more capable over time, which will gradually mitigate the disadvantage. Moreover, we commit to uploading the exact distributions on GitHub, as per the data availability statement, so that the exact probabilities can be shared and thus make them readily available for all who need them.

A topic of future research, proposed by one of the reviewers of this article, is that exact probabilities of the Mann–Whitney U statistic can be obtained analogously relative to the proposed method detailed in Section 2.

## 6. Conclusions

In the early 21th century, a consensus formed within the biomarker literature around the notion that, despite substantial economic resources invested, the scientific community has hitherto failed to produce biomarkers for cancer that translate into clinical use [34,35,36,37,38,39,40]. Among the known pitfalls identified, the use of statistical methods that are unsuitable or improper for the purpose is commonly named among the most meaningful.

The ability to compute exact *p*-values and conduct accurate statistical hypothesis tests of ROC AUC-values, we believe, will facilitate biomarker development and expedite the introduction of IVD devices into clinical use.

## Figures and Tables

**Table 1:** Critical values for two-protein biomarkers. One-sided 99% critical values relative to the null-hypothesis that the biomarker has an AUC-value on par with the colorectal cancer test FIT, which has AUC 0.88, Bonferroni-corrected for the simultaneous testing of 53,352 hypotheses. The numbers of TPs and TNs account for some missing values.

Tumor Type	Number of		Critical Value	Number of
Cohort	TPs	TNs	99%, corrct’d	sign. biom.
Breast	209	800	0.942	0
Colorectal	388	800	0.930	0
Esophagus	45	800	0.978	0
Liver	44	800	0.978	4
Lung	104	800	0.959	0
Ovary	54	372	0.974	14
Pancreas	93	800	0.961	8
Stomach	68	800	0.969	0
Pan-cancer	1004	800	0.919	0

**Table 2:** Two-protein blood biomarkers for liver, ovarian, and pancreatic cancers. Details of the 26 cancer biomarkers that have AUC-values greater than the critical values in Table 1. Proteomic dataset from Ref. [28] and composite biomarkers are formed as per Ref. [31]. Up arrow, ↑, the protein is up-regulated among donors who have cancer relative to the healthy controls. Down arrow, ↓, the protein is down-regulated among cancers relative to controls. ^‡^ Whether it is necessary for positive classification that either or both of the proteins are up or down-regulated. ^§^ The pAUC-value is computed in the interval [0,0.2]. ^¶^ The *p*-value is Bonferroni corrected for simultaneous hypothesis testing of 53,352 biomarkers, under the null-hypothesis that each are on par with the colorectal cancer test FIT, which has AUC 0.88 ([32] p. 21).

Tumour Type	Proteins ^*^	Regulation ^‡^	AUC	pAUC ^§^	*p*-Value ^¶^
Liver	HGF↑ OPN↑	Either	0.983	0.183	$4.33\times 10^{-4}$
	AFP↑ OPN↑	Either	0.982	0.187	$7.85\times 10^{-4}$
	HGF↑ PRL↑	Either	0.979	0.182	$7.03\times 10^{-3}$
	GDF15↑ IL-8↑	Both	0.979	0.179	$8.73\times 10^{-3}$
Ovary	CA-125↑ PRL↑	Either	0.987	0.194	$1.63\times 10^{-6}$
	CA-125↑ TIMP-1↑	Either	0.984	0.186	$6.67\times 10^{-6}$
	CA-125↑ TSP2↓	Either	0.985	0.192	$2.32\times 10^{-5}$
	CA-125↑ IL-6↑	Either	0.982	0.184	$8.12\times 10^{-5}$
	CA125↑ IL-6↑	Both	0.981	0.181	$1.76\times 10^{-4}$
	PRL↑ TIMP-1↑	Either	0.980	0.180	$3.66\times 10^{-4}$
	CA-125↑ GDF15↑	Either	0.978	0.182	$1.22\times 10^{-3}$
	CA-125↑ CEA↓	Either	0.977	0.179	$2.40\times 10^{-3}$
	CA-125↑ OPN↑	Both	0.977	0.182	$2.61\times 10^{-3}$
	CA-125↑ TGF-α↑	Either	0.976	0.184	$2.76\times 10^{-3}$
	CA-125↑ OPN↑	Either	0.976	0.182	$3.16\times 10^{-3}$
	CA-125↑ sFas↓	Either	0.976	0.176	$4.23\times 10^{-3}$
	IL-6↑ sFas↓	Both	0.975	0.178	$6.06\times 10^{-3}$
	CA-125↑ ENG↑	Either	0.974	0.177	$9.03\times 10^{-3}$
Pancreas	CA19-9↑ sErbB2↑	Either	0.971	0.176	$1.88\times 10^{-5}$
	CA19-9↑ GDF15↑	Either	0.967	0.172	$3.68\times 10^{-4}$
	CA19-9↑ OPN↑	Either	0.966	0.171	$8.14\times 10^{-4}$
	CA19-9↑ IL-6↑	Either	0.966	0.172	$1.05\times 10^{-3}$
	CA19-9↑ TIMP-2↑	Either	0.964	0.172	$2.22\times 10^{-3}$
	CA19-9↑ IL-8↑	Either	0.962	0.168	$8.33\times 10^{-3}$
	CA19-9↑ PRL↑	Either	0.962	0.170	$8.89\times 10^{-3}$
	CA19-9↑ HGF↑	Either	0.961	0.173	$9.81\times 10^{-3}$

## Data Availability

Code for the simulation of AUC-value data and AUC-value probability distributions are available on GitHub https://github.com/TSResearchGroupUU/ (accessed on 8 February 2023). Biomarker data available are online via Ref. [28].

## Abbreviations

The following abbreviations are used in this manuscript:

AUC	area under curve
*iid*	independent and identically distributed
IVD	in vitro diagnostic
ROC	receiver operating characteristic
TN	true negative
TP	true positive
